# Evaluation of an automatic segmentation algorithm for definition of head and neck organs at risk

**DOI:** 10.1186/1748-717X-9-173

**Published:** 2014-08-03

**Authors:** David Thomson, Chris Boylan, Tom Liptrot, Adam Aitkenhead, Lip Lee, Beng Yap, Andrew Sykes, Carl Rowbottom, Nicholas Slevin

**Affiliations:** 1 Department of Clinical Oncology, The Christie NHS Foundation Trust, Wilmslow Road, Manchester M20 4BX, UK; 2 Institute of Cancer Sciences, The University of Manchester, Manchester, UK; 3 Department of Medical Physics, The Christie NHS Foundation Trust, Manchester, UK; 4 Clinical Outcomes Unit (medical statistics), The Christie NHS Foundation Trust, Manchester, UK

**Keywords:** Automatic segmentation, SPICE, Head and neck, Organs at risk, Function-sparing IMRT

## Abstract

**Background:**

The accurate definition of organs at risk (OARs) is required to fully exploit the benefits of intensity-modulated radiotherapy (IMRT) for head and neck cancer. However, manual delineation is time-consuming and there is considerable inter-observer variability. This is pertinent as function-sparing and adaptive IMRT have increased the number and frequency of delineation of OARs. We evaluated accuracy and potential time-saving of Smart Probabilistic Image Contouring Engine (SPICE) automatic segmentation to define OARs for salivary-, swallowing- and cochlea-sparing IMRT.

**Methods:**

Five clinicians recorded the time to delineate five organs at risk (parotid glands, submandibular glands, larynx, pharyngeal constrictor muscles and cochleae) for each of 10 CT scans. SPICE was then used to define these structures. The acceptability of SPICE contours was initially determined by visual inspection and the total time to modify them recorded per scan. The Simultaneous Truth and Performance Level Estimation (STAPLE) algorithm created a reference standard from all clinician contours. Clinician, SPICE and modified contours were compared against STAPLE by the Dice similarity coefficient (DSC) and mean/maximum distance to agreement (DTA).

**Results:**

For all investigated structures, SPICE contours were less accurate than manual contours. However, for parotid/submandibular glands they were acceptable (median DSC: 0.79/0.80; mean, maximum DTA: 1.5 mm, 14.8 mm/0.6 mm, 5.7 mm). Modified SPICE contours were also less accurate than manual contours. The utilisation of SPICE did not result in time-saving/improve efficiency.

**Conclusions:**

Improvements in accuracy of automatic segmentation for head and neck OARs would be worthwhile and are required before its routine clinical implementation.

## Background

The accurate definition of organs at risk (OARs) is required to fully exploit the benefits of intensity-modulated radiotherapy (IMRT) for head and neck cancer [1]. However, manual delineation is time-consuming [2]. There is also considerable inter-observer variability; [3-6] which can result in significant differences in radiation dose to OARs [4]. This has implications for: evaluation of radiotherapy plans; interpretation of radiation effects; and meaningful comparisons between treatments. Standardisation is improved by the use of contouring guidelines, multimodality imaging and consensus between experts, but variation in organ delineation remains [3,5,7]. This is of pressing importance with the introduction of both function-sparing and adaptive IMRT, where number and frequency of delineation of OARs are increased.

Following head and neck radiotherapy, adverse late effects are highly prevalent and these impact on both organ function and more general domains of well-being, such as physical, mental and social health [8]. Radiation-induced xerostomia is the most commonly reported grade ≥2 late side effect, which can result in difficulties with speech, swallowing and dental caries [9-11]. Saliva is produced from the major (parotid, submandibular and sublingual) and minor (soft palate, lips, cheeks) salivary glands [12]. The parotid-sparing intensity-modulated versus conventional radiotherapy in head and neck cancer (PARSPORT) trial demonstrated the incidence of grade ≥2 xerostomia one year after treatment was significantly reduced with parotid-sparing IMRT compared to 3D-conformal radiotherapy (38% versus 74%) [9]. One parotid gland should be spared to a mean dose of less than 20Gy or both glands to less than 25Gy [13]. For the submandibular gland, relatively modest reductions in dose (to less than 35Gy) may be of benefit [13].

Swallowing dysfunction is seen in up to half of patients treated with definitive synchronous chemo-radiotherapy and is the most common late grade ≥3 toxicity; the incidence has increased with intensification of treatment including addition of chemotherapy or altered fractionation [14-16]. This adversely affects quality of life, probably to an even greater extent than xerostomia [8,17-20]. The mean radiation doses to the pharyngeal constrictor muscles and supraglottic larynx are significantly associated with late dysphagia [19,21-27]. The volume of the larynx and pharyngeal constrictor muscles that receive a radiation dose ≥60Gy (and where possible ≥50Gy) should be minimised [28].

Permanent and predominantly high frequency sensori-neural hearing loss may occur in 40-60% of patients who receive radiotherapy to areas such as the nasopharynx, para-nasal sinuses and parotid bed [29-31]. This is associated with psychological and cognitive morbidity [32]. The mean dose to the cochlea should be limited to ≤45Gy (or more conservatively ≤35Gy); and when combined with cisplatin, strictly limited [33].

Significant anatomic changes and alteration in dose to target volumes and OARs may occur during a course of head and neck radiotherapy [34-37]. A standard way to detect inter-fraction variation is volumetric imaging using kilovoltage (kV) cone beam computed tomography (CT) imaging. Typically these images are superimposed on the planning CT scan using rigid co-registration. However, this only allows qualitative comparison of similarity in six degrees of freedom, which may not be adequate if the shapes or relative position of target organs and OARs have changed. A potential solution for head and neck structures is the use of automatic segmentation where the planning CT scan and manual contours serve as an atlas and are mapped to the re-planning or cone beam CT scan using a process of deformable registration and voxel-matching [36,38-41]. This would facilitate calculation of changes in doses to the target volumes and OARs; [42] information that could be used to determine whether adaptive re-planning is required [34,43-45].

Smart Probabilistic Image Contouring Engine (SPICE) is an automated commercially available algorithm, which combines an atlas-based and model-based approach to segmentation of head and neck lymph node levels and OARs [46]. The atlas was initially derived from expert ‘ground truth’ contours. The automatic segmentation process employs multiple-steps of deformable image registration. First, low-dimensional non rigid transformation maps the model landmarks (or mean organ positions) into the image, which accounts for any large displacements (atlas-based step). Second, there is density-based registration where each voxel is included or excluded from a structure depending on its intensity (grey-scale step) i.e., functionality is limited to CT scans. Third, a model-based segmentation approach is applied where organ models (‘meshes’) that have been created from averaged manual expert segmentations adapts and refines the structure (shape model-based step). This mesh evolution can be considered as being ‘driven by the grey-scale and constrained by the shape model’ [47].

This study aims to evaluate accuracy and time-saving of SPICE to define OARs for salivary-, swallowing- and cochlea-sparing IMRT.

## Methods

Ten radiotherapy planning CT scans were selected where the OARs of interest were not distorted by tumour or artefact (treatment planning system, Pinnacle³ version 9.4). Five clinicians (four Consultants/Attending Physicians and one Fellow) recorded for each scan the time to manually delineate the parotid and submandibular glands, larynx (supraglottic and glottic larynx defined as one structure), pharyngeal constrictor muscles (superior, middle, inferior pharyngeal constrictor muscles and cricopharyngeus muscle defined as one structure) and cochleae according to a locally agreed protocol based on published guidelines (‘manual’ contours) [14,48,49]. SPICE was then used to define these structures (‘SPICE’ contours). Each clinician determined by visual inspection the acceptability of SPICE contours for each structure and the total time to modify these for each scan (‘modified SPICE’ contours). The modified SPICE contours represent the *utilisation* of SPICE in clinical practice (clinician review and modification). These also demonstrate introduction of bias by automatic segmentation (in the absence of bias, modified and manual contours should ideally match).

**Figure 1:**
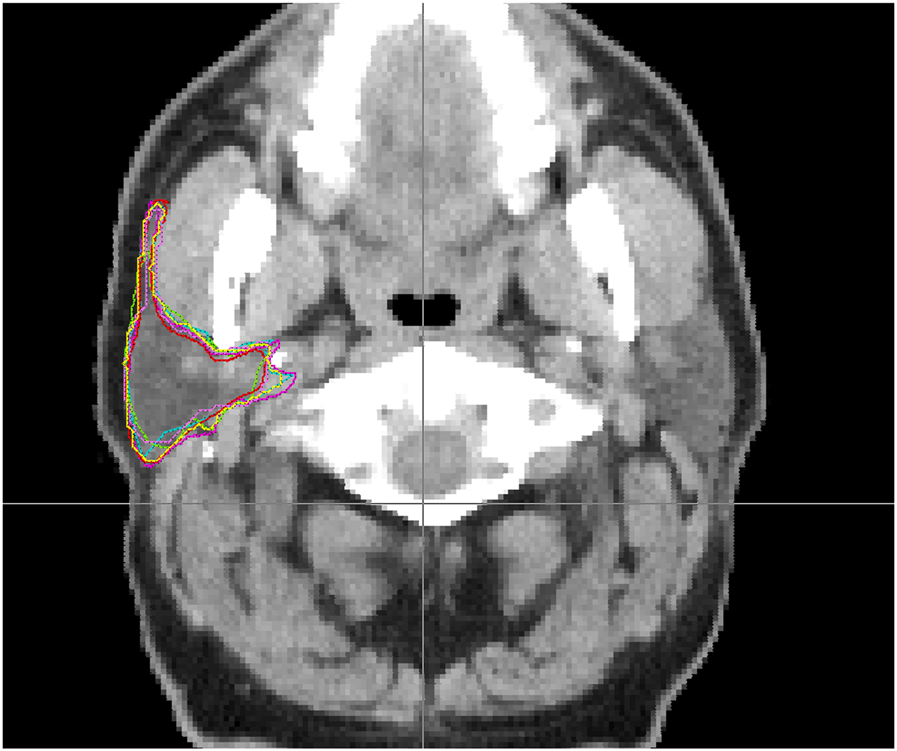
Right parotid gland defined by five manual (multiple colours) and one STAPLE (yellow) contour (one transverse CT slice shown).

The Simultaneous Truth and Performance Level Estimation (STAPLE) algorithm employs a probability map to create a ‘best fit’ from a collection of contours (Figure 1) [50]. The STAPLE algorithm created a reference standard from all clinician manual contours (‘STAPLE’ contours). The manual, SPICE and modified SPICE contours were compared to STAPLE by: Dice similarity coefficient (DSC) and mean/maximum distance to agreement (DTA). The DSC is a statistical measure of spatial overlap between two structures. It is defined as 2x intersection volume/total sum of volumes and normalises the degree of intersection from 0 (no overlap) to 1 (perfect overlap), with good agreement defined as >0.7-0.8 [41,51,52]. DTA is a geometrical parameter that measures the per voxel shortest distance from the surface of one structure to another, ideal = 0 mm. Paired structures (parotid glands, submandibular glands and cochleae) were considered together. For the parotid and submandibular glands, SPICE generated three contours (‘1’ , ‘2’ or ‘3’), which were each based on different ‘ground truth’ data [53]. Comparisons between these and STAPLE for all 10 patients were made to determine the most accurate, for subsequent use and evaluation. The study was conducted with appropriate local R&D approval.

Statistical comparisons using multiple linear regression analysis (to control for possible individual patient/scan or clinician confounding factors) were made between mean values of all matrices for: SPICE against STAPLE versus manual against STAPLE (to determine the accuracy of SPICE); and modified SPICE against STAPLE versus manual against STAPLE (to determine the accuracy of modified SPICE i.e., the utilisation of SPICE). As a further measure of accuracy, SPICE was compared with the most discordant clinician contours (determined against STAPLE and by ranking of clinicians) for each structure measured by DSC and DTA, using the Wilcoxon signed rank test. The total times to manual versus modify SPICE contours for all structures and clinicians were compared using Student’s paired *t*-test (to determine efficiency in the utilisation of SPICE). Significance was assessed at the p < 0.05 level.

## Results

### Accuracy of SPICE

**Figure 2:**
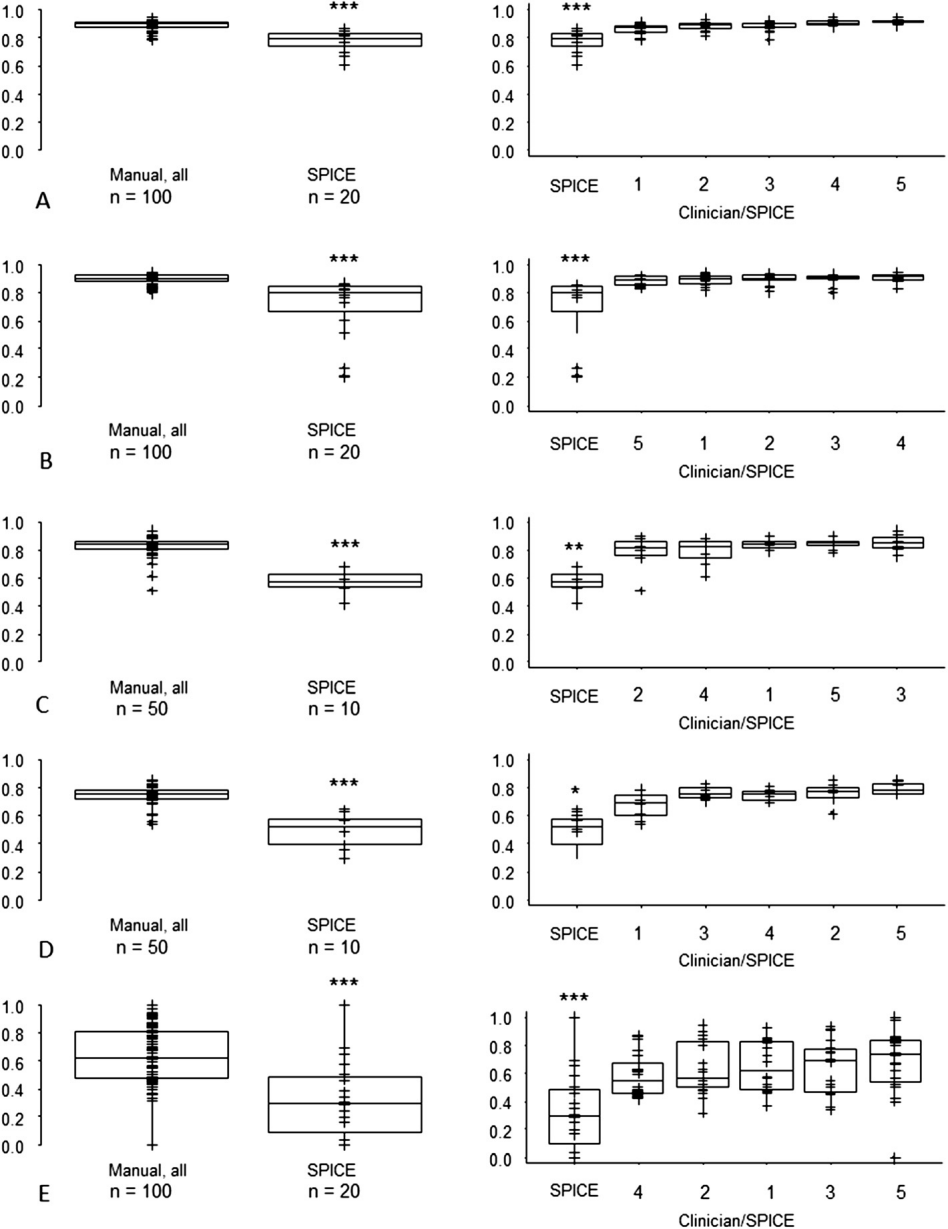
**Dice similarity coefficient - SPICE against STAPLE compared with: (i) all manual contours against STAPLE (left-side graphs); (ii) individual clinicians manual contours against STAPLE (right-side graphs, statistical comparisons shown between most discordant clinician contours against STAPLE versus SPICE against STAPLE) for A. parotid glands, B. submandibular glands, C. larynx, D. pharyngeal constrictor muscles, E. cochleae.** *p < 0.05, **p < 0.01, ***p < 0.001. Abbreviations: n, total number of manual or SPICE contours (for paired organs, two per scan).

**Figure 3:**
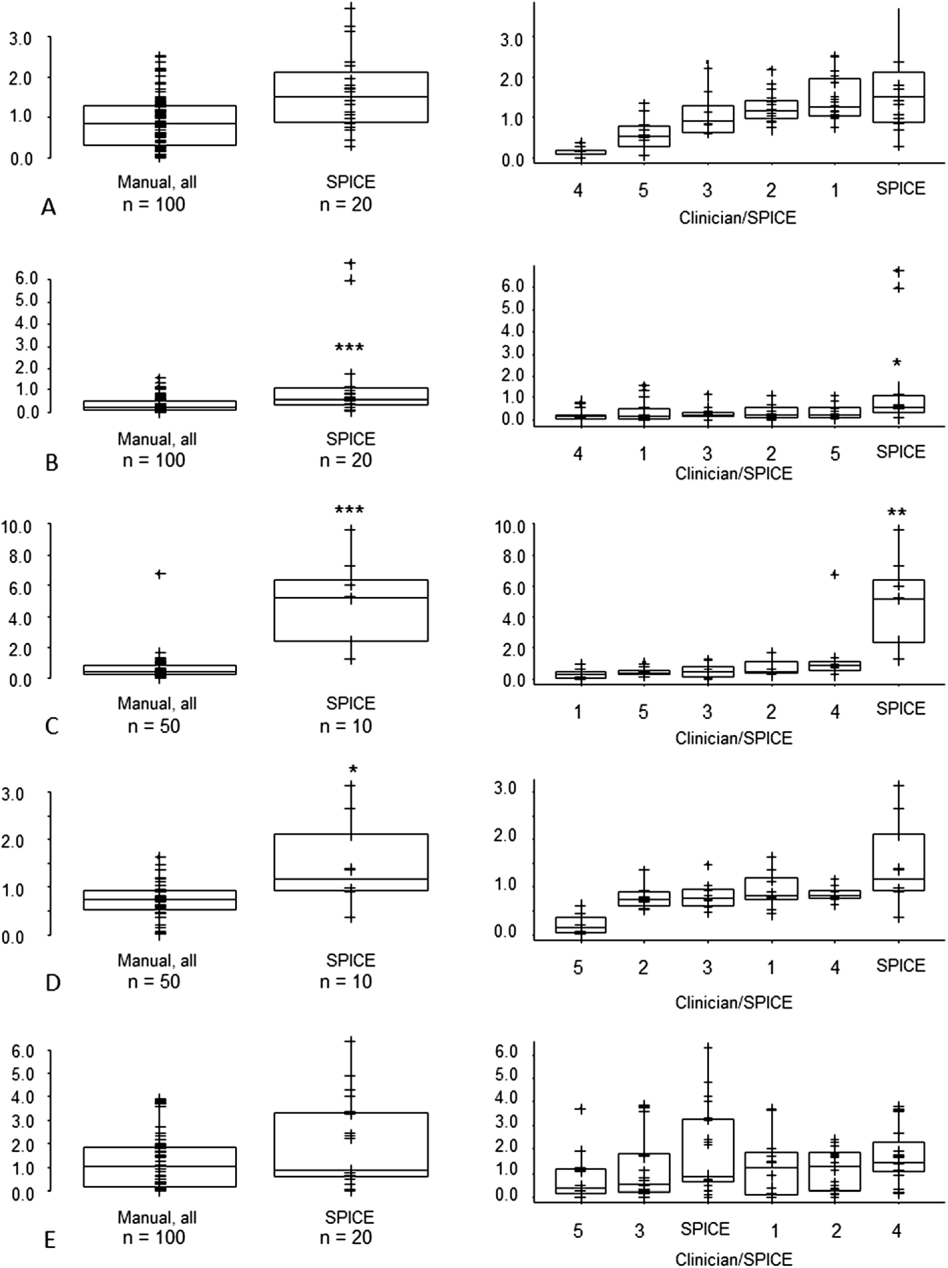
**Mean Distance to Agreement (mm) - SPICE against STAPLE compared with: (i) all manual contours against STAPLE (left-side graphs); (ii) individual clinicians manual contours against STAPLE (right-side graphs, statistical comparisons shown between most discordant clinician contours against STAPLE versus SPICE against STAPLE) for A. parotid glands, B. submandibular glands, C. larynx, D. pharyngeal constrictor muscles, E. cochleae.** *p < 0.05, **p < 0.01, ***p < 0.001. Abbreviations: n, total number of manual or SPICE contours (for paired organs, two per scan).

**Figure 4:**
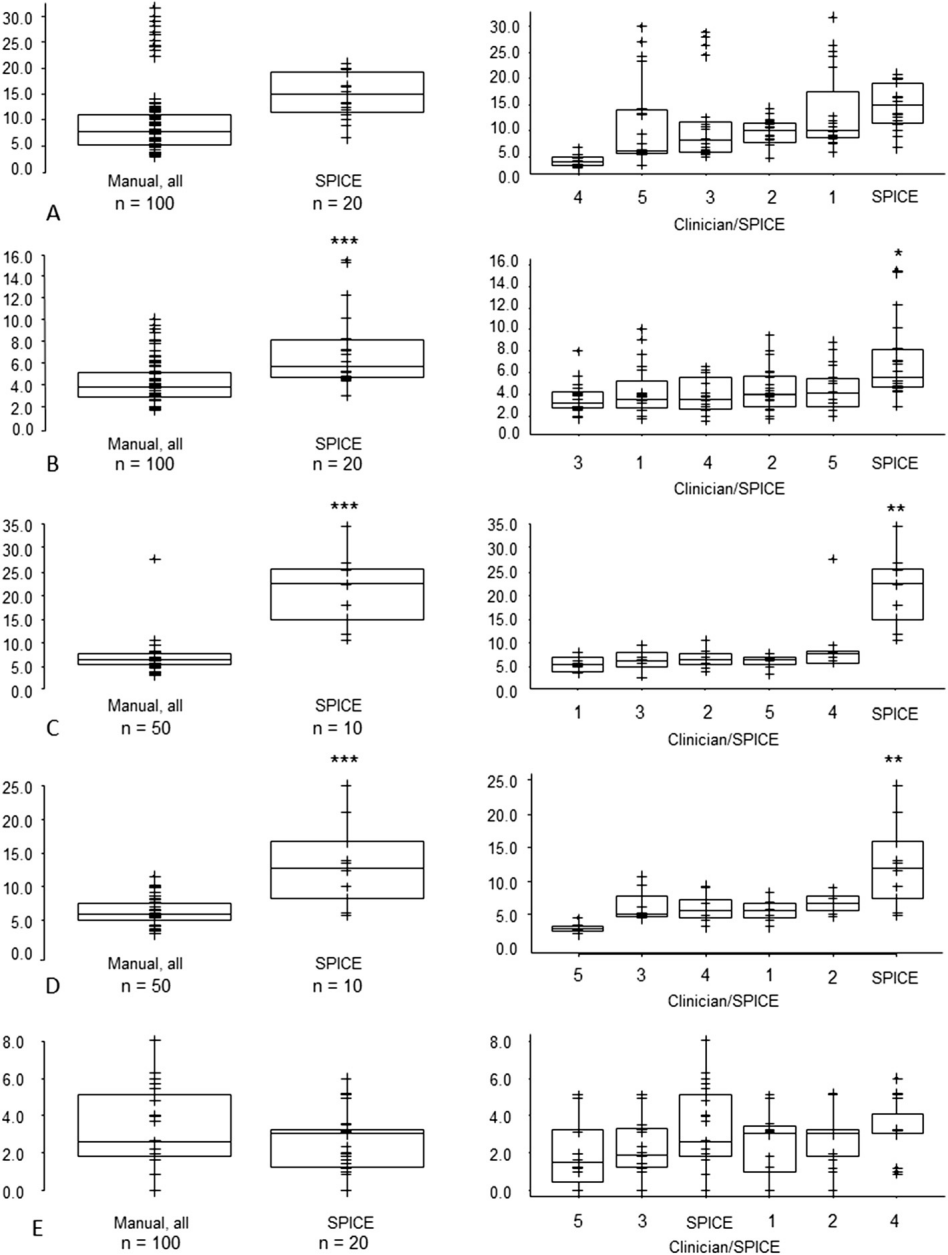
**Maximum Distance to Agreement (mm) - SPICE against STAPLE compared with: (i) all manual contours against STAPLE (left-side graphs); (ii) individual clinicians manual contours against STAPLE (right-side graphs, statistical comparisons shown between most discordant clinician contours against STAPLE versus SPICE against STAPLE) for A. parotid glands, B. submandibular glands, C. larynx, D. pharyngeal constrictor muscles, E. cochleae.** *p < 0.05, **p < 0.01, ***p < 0.001. Abbreviations: n, total number of manual or SPICE contours (for paired organs, two per scan).

SPICE submandibular gland ‘1’ and parotid gland ‘2’ contours demonstrated best concordance with STAPLE (Table 1) and were used in subsequent comparisons.The mean DSCs were significantly reduced for SPICE contours compared with manual for all structures (Figure 2). All SPICE contours were inferior to the most discordant manual contours (Figure 2). However, for parotid and submandibular glands SPICE contours, the respective median and interquartile ranges for DSCs were 0.79 (0.74, 0.83) and 0.80 (0.70, 0.85), suggesting acceptability for these structures. The mean and maximum DTAs for SPICE contours and manual were similar for parotid glands and cochleae but statistically significantly worse for submandibular glands, larynx and pharyngeal constrictor muscles (Figures 3 and 4). Similarly, except for the parotid glands and cochleae, the SPICE contours mean and maximum DTAs were inferior to the most discordant clinician manual contours. However, for submandibular glands, the respective median and interquartile ranges for mean and maximum DTAs were relatively minor: 0.6 mm (0.4-1.0) and 5.6 mm (4.7-8.2 mm).

**Table 1:** SPICE version 1, 2 and 3 against STAPLE for definition of parotid and submandibular glands; mean/median values shown

**SPICE contours n = 20**	**DSC**	**mean DTA (mm)**	**max DTA (mm)**
Parotid Glands			
1	0.77/0.79	2.2/1.9	19.0/17.4
2	0.78/0.79	1.6/1.5	14.7/14.8
3	0.72/0.75	3.3/2.5	21.5/19.8
Submandibular Glands			
1	0.70/0.80	1.5/0.6	7.2/5.7
2	0.67/0.78	2.0/0.4	8.2/5.6
3	0.64/0.68	3.0/1.0	11.4/8.0

Abbreviations: *DSC* Dice similarity coefficient, *DTA* distance to agreement, *max* maximum, *mm* millimetres, *n* number of SPICE 1, 2 or 3 contours for parotid or submandibular glands (10 CT scans, two of each structure per scan) Parotid gland ‘2’ and submandibular gland ‘1’ SPICE contours were taken forward for subsequent investigation.

### Utilisation of SPICE

**Figure 5:**
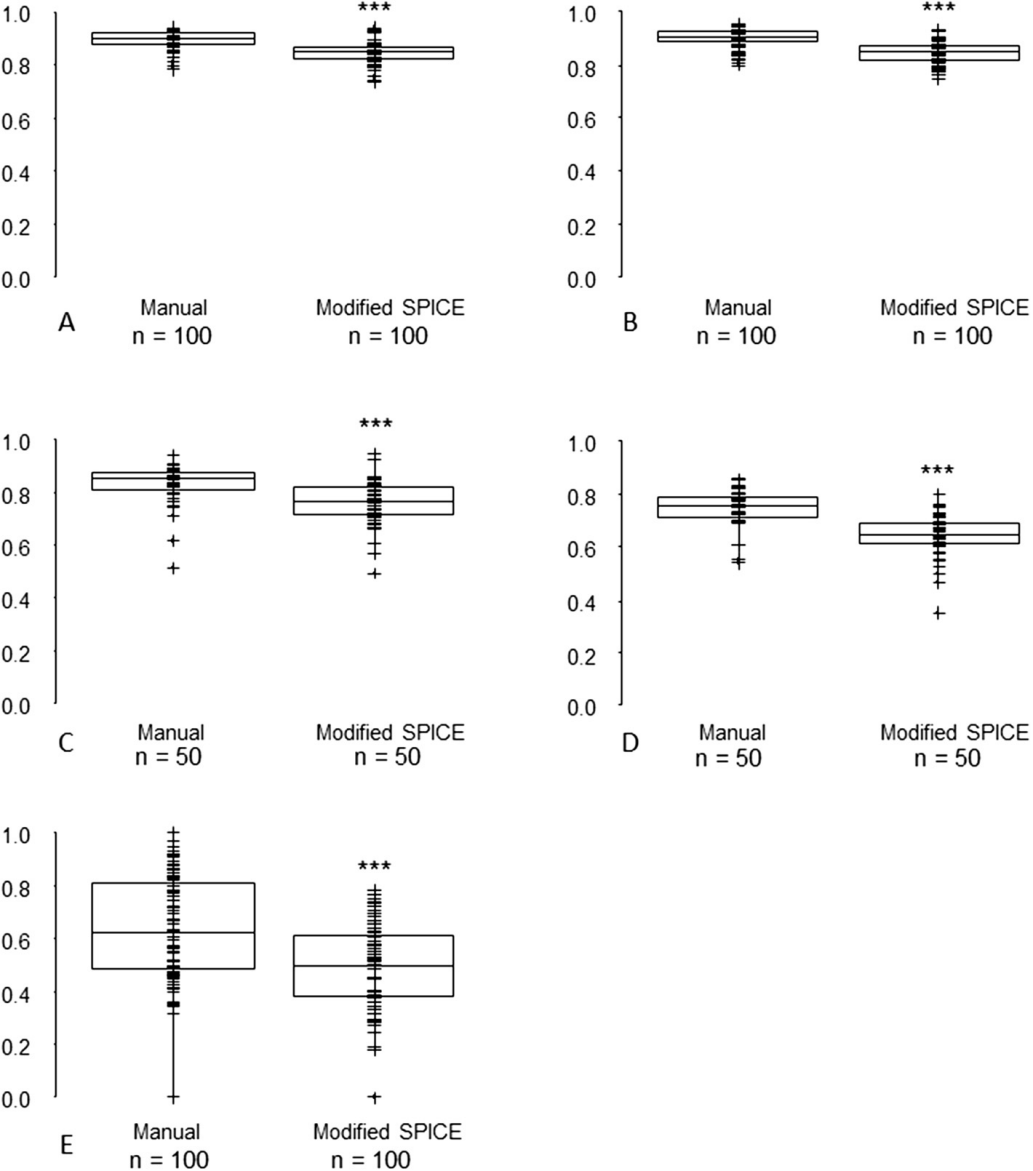
**Dice similarity coefficient – Modified SPICE against STAPLE compared with all manual contours against STAPLE for A. parotid glands, B. submandibular glands, C. larynx, D. pharyngeal constrictor muscles, E. cochleae.** *p < 0.05, **p < 0.01, ***p < 0.001. Abbreviations: n, total number of manual or modified SPICE contours (for paired organs, two per scan).

**Figure 6:**
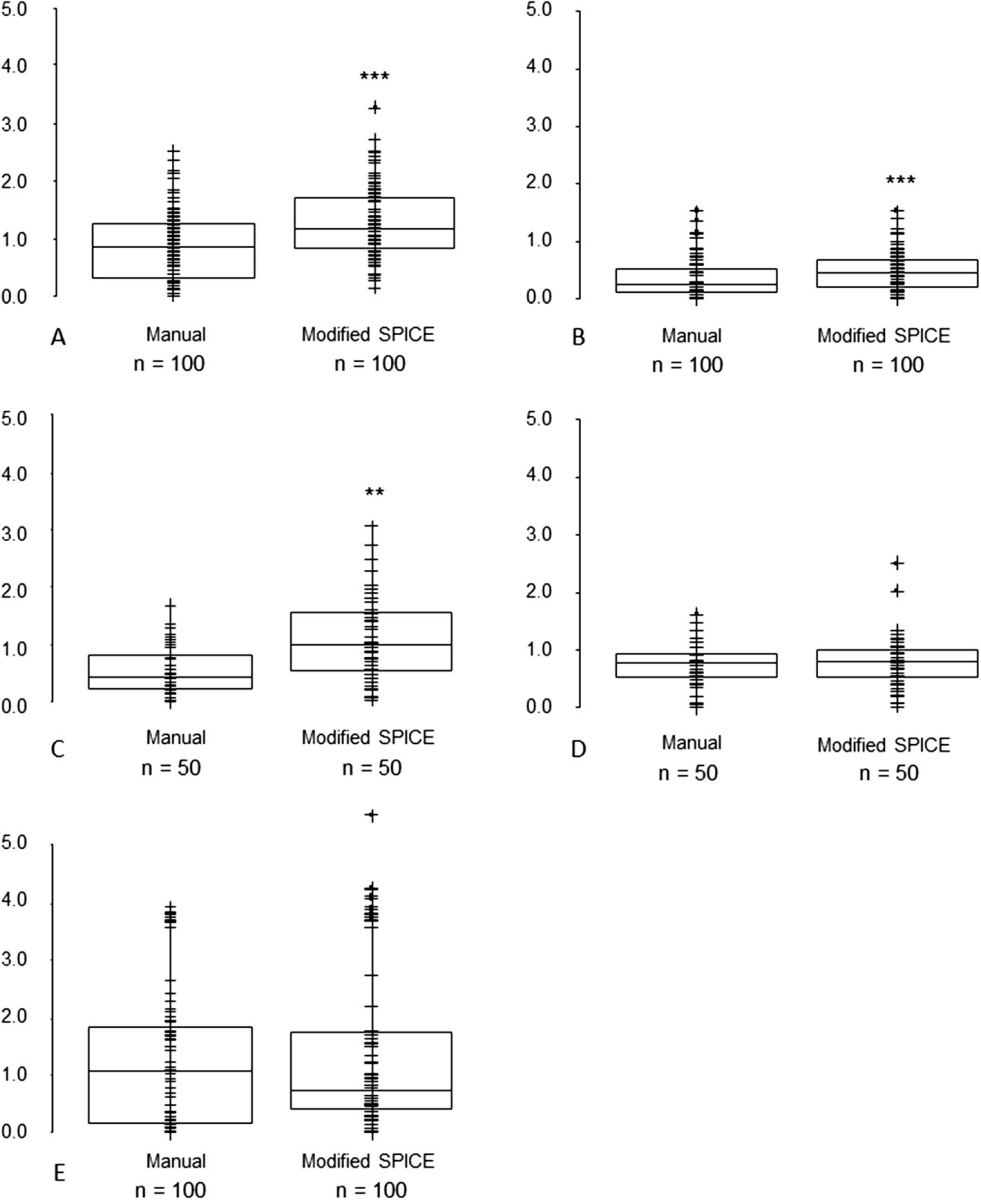
**Mean Distance to Agreement (mm) – Modified SPICE against STAPLE compared with all manual contours against STAPLE for A. parotid glands, B. submandibular glands, C. larynx, D. pharyngeal constrictor muscles, E. cochleae.** *p < 0.05, **p < 0.01, ***p < 0.001. Abbreviations: n, total number of manual or modified SPICE contours (for paired organs, two per scan).

**Figure 7:**
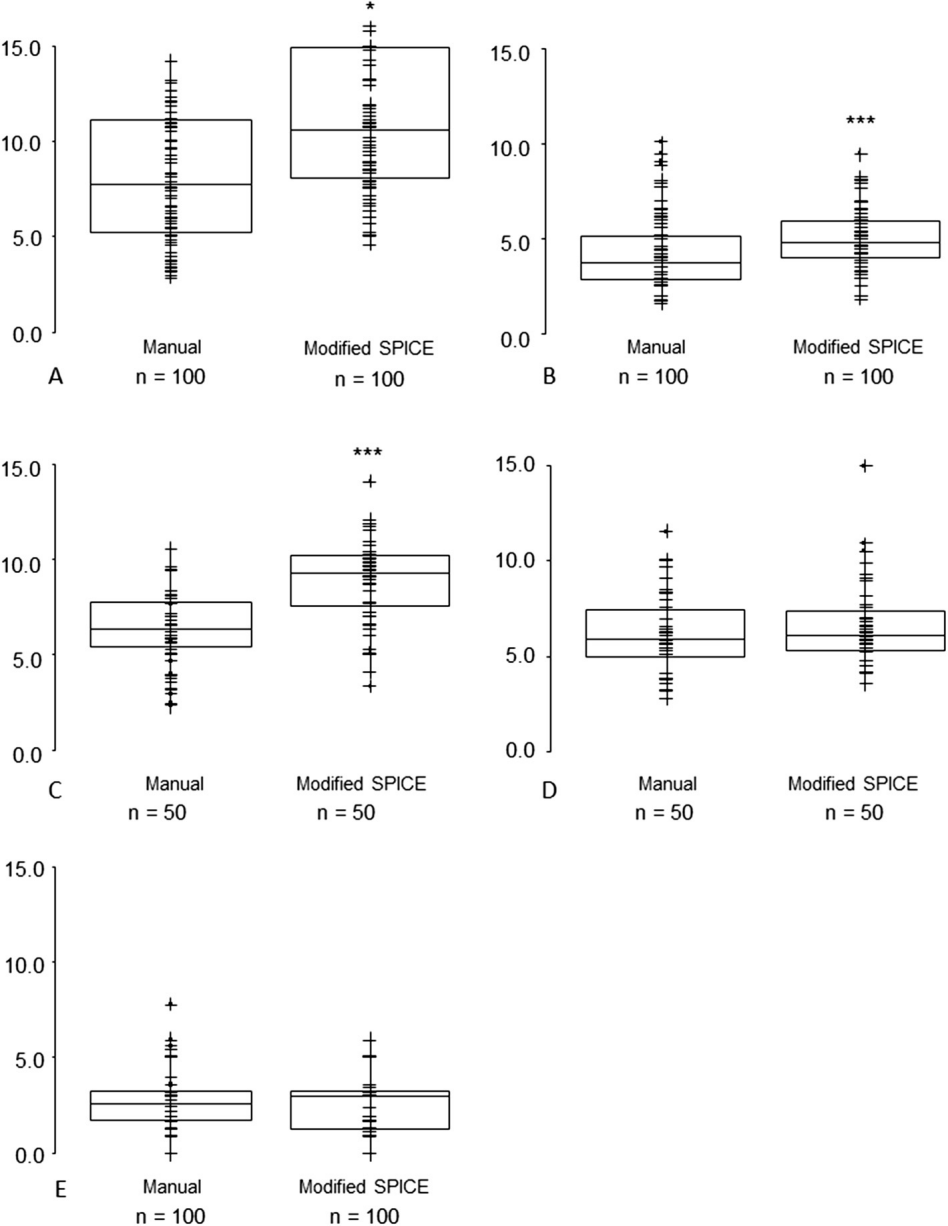
**Maximum Distance to Agreement (mm) – Modified SPICE against STAPLE compared with all manual contours against STAPLE for A. parotid glands, B. submandibular glands, C. larynx, D. pharyngeal constrictor muscles, E. cochleae.** *p < 0.05, **p < 0.01, ***p < 0.001. Abbreviations: n, total number of manual or modified SPICE contours (for paired organs, two per scan).

The total proportions of SPICE contours determined by visual inspection not to require alteration were: parotid glands (17%), submandibular glands (41%), larynx (8%), pharyngeal constrictor muscles (4%), and cochleae (28%). The mean DSCs were significantly reduced for modified SPICE contours compared with manual for all structures (Figure 5). However, the respective median and interquartile ranges for modified SPICE DSCs for parotid glands, submandibular glands and larynx were: 0.85 (0.83, 0.86), 0.85 (0.82, 0.87), and 0.76 (0.72, 0.82), which represented good agreement. The mean and maximum DTAs for modified SPICE contours compared with manual were similar for the pharyngeal constrictor muscles and cochleae but significantly worse for parotid glands, submandibular glands and larynx (Figures 6 and 7). For these three structures, the respective median and interquartile ranges for the mean/maximum DTAs were 1.2 mm (0.8 mm-1.7 mm)/10.6 mm (8.0 mm-14.8 mm), 0.4 mm (0.2 mm-0.7 mm)/4.8 mm (4.0 mm-5.9 mm), 1.0 mm (0.6 mm-1.6 mm)/9.3 mm (7.6-10.2), representing relatively minor differences for submandibular glands.

### Efficiency in utilisation of SPICE

**Figure 8:**
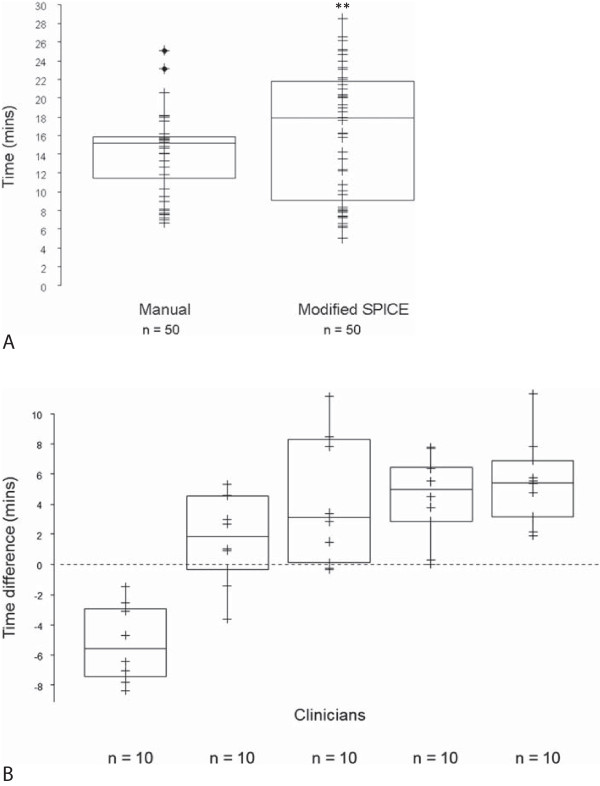
**Efficiency in utilisation of SPICE - A. Total time per scan for all clinicians to manual and modify SPICE contours; and B. Time differences per scan between modified SPICE contours compared with manual for each clinician.** (positive values: increase in time to modified versus manual contours); **p< 0.01. Abbreviations: n, total number of CT scans.

The respective per scan overall mean times for manual and modified SPICE contours were 14.0 and 16.2 minutes (difference, 15.7%) (Figure 8). Only one out of five clinicians showed a mean reduction in per scan overall time to modify SPICE contours compared with manual.

## Discussion

This study showed that for head and neck OARs: (i) SPICE contours were less accurate than manual contours, but acceptable for the definition of parotid and submandibular glands; (ii) modified SPICE contours remained inferior to manual contours; and (iii) the utilisation of SPICE compared with manual delineation did not result in time-saving/improve efficiency.

Automatic segmentation to define selected head and neck OARs may reduce inter-observer variability [54,55]. Chao *et al* compared for two CT scans and eight clinicians, manual and automatic modified contours for delineation of the clinical target volume as well as parotid glands, spinal cord, brainstem and (for one scan) the optic apparatus [54]. For the OARs, inter-observer variability was significantly reduced for modified compared with manual contours. This was associated with a mean time saving of 26%-47%, which depended on experience of the oncologist. In a subsequent study, the *ISOgray* atlas-based auto-segmentation algorithm was evaluated for definition of the brainstem, parotid glands and mandible [55]. The study was conducted at 2 centres, where a total of 3 clinicians either manually delineated (2 clinicians, 3 scans each) or modified automated contours (1 clinician, 7 scans); for only one scan were both manual and modified contours defined. The mean DSCs for all organs were 0.68 and 0.82 for manual and modified contours, respectively; and the sensitivity and specificity for manual versus modified contours were 63%-91% and 60%-80% versus 63-91% and 89-98%, respectively. These results suggested reduced inter-observer variability for modified contours compared with manual. However, while demonstration of reduced inter-observer variability is important, it is not sufficient, because there is potential introduction of bias and systematic errors.

The updated *Brainlab* automated segmentation algorithm, which employs atlas-based and deformable registration, was assessed for accuracy of definition of neck nodal regions and selected head and neck OARs [56]. In 10 ‘ideal’ cases without neck nodes on at least one side, the ipsilateral parotid gland, spinal cord and brainstem were contoured; and in 10 cases with neck node involvement both parotid glands, submandibular glands, spinal cord, brainstem and mandible were defined. One clinician manually contoured and then modified the automatic contours for each scan/patient. The automatic and modified contours were compared with manual contours using the DSC as well as mean and maximum DTA. The spinal cord and mandible contours were not included in the analysis because the automatic contours did not require modification, except for mandible in one case. For the second group of 10 cases, the OARs were considered together. The authors found that except for spinal cord, the automatic contours systematically required some modification, with resultant improvement in DSC and DTA measures. There was increased efficiency in definition of OARs with a reduction in mean time to manual compared with modified contours from 11.2 minutes to 4.5 minutes (60%) and 16.4 to 6.3 minutes (62%), in respective groups. This time-saving is partly due to the automatic contours for spinal cord, brainstem and mandible requiring no or little modification.

Clinical validation of a multiple-subject atlas-based autosegmentation tool was performed by measuring the DSC and mean DTA for manual contours (outlined by one of 10 clinicians and agreed by an expert panel) and modified contours (outlined by one of two clinicians) for neck levels, parotid and submandibular glands in 12 patients [57]. For manual versus automatic contours, the respective DSC/mean DTA for parotid and submandibular glands were 0.80/2.3 mm and 0.72/1.6 mm. For manual versus modified automatic contours, the respective DSC/mean DTA for parotid and submandibular glands were 0.81/2.1 mm and 0.77/1.2 mm.

We found that SPICE automatic contours were less accurate/inferior to manual contours for all investigated structures, but acceptable for the parotid and submandibular glands. For the parotid and submandibular glands, the DSCs were satisfactory; [41,52] for parotid glands, the mean and maximum DTAs were similar to manual contours and for submandibular glands, the differences were relatively minor. The modification of automatic contours improved accuracy but remained inferior to manual contours and did not result in time-saving. There are a number of possible reasons for these findings. First, the processes of automatic segmentation, both grey-scale and model-based are limited by insensitivity to boundary or edge detection [47]. This is important because the differences in attenuation between soft tissues are often small and the shapes of organs divergent. The computer-based algorithms do not account for nuances in the honed technique of the expert manual contourer. Second, while there are published delineation guidelines for OARs, there is no agreed international consensus, especially for definition of the larynx and pharyngeal constrictor muscles [14,48]. The SPICE atlas may have been developed from dissimilar ‘ground truth’ contours. Where available, an alternative investigational strategy would be to adapt the local contouring protocol to that used to define the atlas contours [58]. Third, to produce tightly conformed volumes, relatively small alterations in automatic contours may be required, which are time-consuming. The modification process is then less efficient than manual delineation, where techniques such as interpolation between CT slice levels may be used.

Whether differences between manual, automatic or modified contours result in clinically relevant alterations in measured doses to OARs is uncertain. This will partly depend on proximity of normal structures to the treatment volume and the dose gradient. In this study, the target volumes were not defined. This may have influenced the low percentage of OARs determined by visual inspection not to require alteration i.e., clinicians only considered the conformity of automatic contours to normal structures rather than clinical relevance or requirement for this.

This study represents an independent clinical evaluation of automatic segmentation using SPICE and its utilisation for head and neck OARs. It determined the accuracy of SPICE by comparison against a reference standard created using STAPLE, for five head and neck OARs important in function-sparing IMRT. Future work should evaluate automatic segmentation in the presence of distortion by tumour or artefact e.g., dental amalgam; and determine the variation in measured dose to OARs between manual, automatic and modified contours.

## Conclusion

For the investigated head and neck OARs, SPICE automatic segmentations were less accurate than manual contours. However, these were acceptable for the definition of parotid and submandibular glands. The modification of SPICE contours improved accuracy, but these remained inferior to manual contours and the process did not result in time-saving. Improvements in automatic segmentation of head and neck OARs would be worthwhile and are required before routine clinical implementation.

## Abbreviations

OARs: Organs at risk; IMRT: Intensity-modulated radiotherapy; SPICE: Smart Probabilistic Image Contouring Engine; STAPLE: Simultaneous Truth and Performance Level Estimation; DSC: Dice Similarity Coefficient; DTA: Distance to agreement; PARSPORT: Parotid-sparing intensity modulated versus conventional radiotherapy in head and neck cancer; Gy: Gray; kV: kilovoltage; CT: Computed tomography.

## Competing interests

The authors declare that they have no competing interests.

## Authors’ contributions

DT designed and coordinated the study, participated in contouring, analysed part of the data, interpreted data, drafted the manuscript. CB performed STAPLE and volume overlap measurements. TL analysed the data. AA provided the DTA algorithm and helped with volume overlap measurements. LL, BY, AJS, NJS participated in contouring. CR/NJS conceived the study, participated in its design and coordination and helped draft the manuscript. All authors read and approved the final manuscript.

## References

[B1] Stapleford LJ Lawson JD Perkins C Edelman S Davis L McDonald MW Waller A Schreibmann E Fox T Evaluation of automatic atlas-based lymph node segmentation for head-and-neck cancer Int J Radiat Oncol Biol Phys 2010 77 959 966 20231069 10.1016/j.ijrobp.2009.09.023

[B2] Miles EA Clark CH Urbano MT Bidmead M Dearnaley DP Harrington KJ A'Hern R Nutting CM The impact of introducing intensity modulated radiotherapy into routine clinical practice Radiother Oncol 2005 77 241 246 16298002 10.1016/j.radonc.2005.10.011

[B3] Geets X Daisne JF Arcangeli S Coche E De Poel M Duprez T Nardella G Gregoire V Inter-observer variability in the delineation of pharyngo-laryngeal tumor, parotid glands and cervical spinal cord: comparison between CT-scan and MRI Radiother Oncol 2005 77 25 31 15919126 10.1016/j.radonc.2005.04.010

[B4] Nelms BE Tome WA Robinson G Wheeler J Variations in the contouring of organs at risk: test case from a patient with oropharyngeal cancer Int J Radiat Oncol Biol Phys 2012 82 368 378 21123004 10.1016/j.ijrobp.2010.10.019

[B5] Brouwer CL Steenbakkers RJ van den Heuvel E Duppen JC Navran A Bijl HP Chouvalova O Burlage FR Meertens H Langendijk JA van’t Veld AA 3D variation in delineation of head and neck organs at risk Radiat Oncol 2012 7 32 22414264 10.1186/1748-717X-7-32 PMC3337234

[B6] O'Daniel JC Rosenthal DI Garden AS Barker JL Ahamad A Ang KK Asper JA Blanco AI de Crevoisier R Holsinger FC Patel CB Schwartz DL Wang H Dong L The effect of dental artifacts, contrast media, and experience on interobserver contouring variations in head and neck anatomy Am J Clin Oncol 2007 30 191 198 17414470 10.1097/01.coc.0000256704.58956.45

[B7] Mukesh M Benson R Jena R Hoole A Roques T Scrase C Martin C Whitfield GA Gemmill J Jefferies S Interobserver variation in clinical target volume and organs at risk segmentation in post-parotidectomy radiotherapy: Can segmentation protocols help? Br J Radiol 2012 85 e530 e536 22815423 10.1259/bjr/66693547 PMC3587102

[B8] Langendijk JA Doornaert P Verdonck-de Leeuw IM Leemans CR Aaronson NK Slotman BJ Impact of late treatment-related toxicity on quality of life among patients with head and neck cancer treated with radiotherapy J Clin Oncol 2008 26 3770 3776 18669465 10.1200/JCO.2007.14.6647

[B9] Nutting CM Morden JP Harrington KJ Urbano TG Bhide SA Clark C Miles EA Miah AB Newbold K Tanay M Adab F Jefferies SJ Scrase C Yap BK A'Hern RP Sydenham MA Emson M Hall E Group Ptm Parotid-sparing intensity modulated versus conventional radiotherapy in head and neck cancer (PARSPORT): a phase 3 multicentre randomised controlled trial Lancet Oncol 2011 12 127 136 21236730 10.1016/S1470-2045(10)70290-4 PMC3033533

[B10] Wijers OB Levendag PC Braaksma MM Boonzaaijer M Visch LL Schmitz PI Patients with head and neck cancer cured by radiation therapy: a survey of the dry mouth syndrome in long-term survivors Head Neck 2002 24 737 747 12203798 10.1002/hed.10129

[B11] Guchelaar HJ Vermes A Meerwaldt JH Radiation-induced xerostomia: pathophysiology, clinical course and supportive treatment Support Care Cancer 1997 5 281 288 9257424 10.1007/s005200050075

[B12] Hoebers F Yu E Eisbruch A Thorstad W O'Sullivan B Dawson LA Hope A A pragmatic contouring guideline for salivary gland structures in head and neck radiation oncology: The MOIST target Am J Clin Oncol 2013 36 70 76 22237147 10.1097/COC.0b013e31823a538e

[B13] Deasy JO Moiseenko V Marks L Chao KS Nam J Eisbruch A Radiotherapy dose-volume effects on salivary gland function Int J Radiat Oncol Biol Phys 2010 76 S58 S63 20171519 10.1016/j.ijrobp.2009.06.090 PMC4041494

[B14] Christianen ME Langendijk JA Westerlaan HE van de Water TA Bijl HP Delineation of organs at risk involved in swallowing for radiotherapy treatment planning Radiother Oncol 2011 101 394 402 21664711 10.1016/j.radonc.2011.05.015

[B15] Goguen LA Posner MR Norris CM Tishler RB Wirth LJ Annino DJ Gagne A Sullivan CA Sammartino DE Haddad RI Dysphagia after sequential chemoradiation therapy for advanced head and neck cancer Otolaryngol Head Neck Surg 2006 134 916 922 16730530 10.1016/j.otohns.2006.02.001

[B16] Rosenthal DI Lewin JS Eisbruch A Prevention and treatment of dysphagia and aspiration after chemoradiation for head and neck cancer J Clin Oncol 2006 24 2636 2643 16763277 10.1200/JCO.2006.06.0079

[B17] List MA Siston A Haraf D Schumm P Kies M Stenson K Vokes EE Quality of life and performance in advanced head and neck cancer patients on concomitant chemoradiotherapy: a prospective examination J Clin Oncol 1999 17 1020 1028 10071297 10.1200/JCO.1999.17.3.1020

[B18] Nguyen NP Frank C Moltz CC Vos P Smith HJ Karlsson U Dutta S Midyett A Barloon J Sallah S Impact of dysphagia on quality of life after treatment of head-and-neck cancer Int J Radiat Oncol Biol Phys 2005 61 772 778 15708256 10.1016/j.ijrobp.2004.06.017

[B19] Jensen K Lambertsen K Grau C Late swallowing dysfunction and dysphagia after radiotherapy for pharynx cancer: Frequency, intensity and correlation with dose and volume parameters Radiother Oncol 2007 85 74 82 17673322 10.1016/j.radonc.2007.06.004

[B20] Hunter KU Schipper M Feng FY Lyden T Haxer M Murdoch-Kinch CA Cornwall B Lee CS Chepeha DB Eisbruch A Toxicities affecting quality of life after chemo-IMRT of oropharyngeal cancer: Prospective study of patient-reported, observer-rated, and objective outcomes Int J Radiat Oncol Biol Phys 2013 85 935 940 23040224 10.1016/j.ijrobp.2012.08.030 PMC3556374

[B21] Dirix P Abbeel S Vanstraelen B Hermans R Nuyts S Dysphagia after chemoradiotherapy for head-and-neck squamous cell carcinoma: Dose-effect relationships for the swallowing structures Int J Radiat Oncol Biol Phys 2009 75 385 392 19553033 10.1016/j.ijrobp.2008.11.041

[B22] Dornfeld K Simmons JR Karnell L Karnell M Funk G Yao M Wacha J Zimmerman B Buatti JM Radiation doses to structures within and adjacent to the larynx are correlated with long-term diet- and speech-related quality of life Int J Radiat Oncol Biol Phys 2007 68 750 757 17418971 10.1016/j.ijrobp.2007.01.047

[B23] Schwartz DL Hutcheson K Barringer D Tucker SL Kies M Holsinger FC Ang KK Morrison WH Rosenthal DI Garden AS Dong L Lewin JS Candidate dosimetric predictors of long-term swallowing dysfunction after oropharyngeal intensity-modulated radiotherapy Int J Radiat Oncol Biol Phys 2010 78 1356 1365 20646872 10.1016/j.ijrobp.2009.10.002 PMC4034521

[B24] Caglar HB Tishler RB Othus M Burke E Li Y Goguen L Wirth LJ Haddad RI Norris CM Court LE Aninno DJ Posner MR Allen AM Dose to larynx predicts for swallowing complications after intensity-modulated radiotherapy Int J Radiat Oncol Biol Phys 2008 72 1110 1118 18468812 10.1016/j.ijrobp.2008.02.048

[B25] Caudell JJ Schaner PE Desmond RA Meredith RF Spencer SA Bonner JA Dosimetric factors associated with long-term dysphagia after definitive radiotherapy for squamous cell carcinoma of the head and neck Int J Radiat Oncol Biol Phys 2010 76 403 409 19467801 10.1016/j.ijrobp.2009.02.017

[B26] Levendag PC Teguh DN Voet P van der Est H Noever I de Kruijf WJ Kolkman-Deurloo IK Prevost JB Poll J Schmitz PI Heijmen BJ Dysphagia disorders in patients with cancer of the oropharynx are significantly affected by the radiation therapy dose to the superior and middle constrictor muscle: a dose-effect relationship Radiother Oncol 2007 85 64 73 17714815 10.1016/j.radonc.2007.07.009

[B27] Christianen ME Schilstra C Beetz I Muijs CT Chouvalova O Burlage FR Doornaert P Koken PW Leemans CR Rinkel RN de Bruijn MJ de Bock GH Roodenburg JL van der Laan BF Slotman BJ Verdonck-de Leeuw IM Bijl HP Langendijk JA Predictive modelling for swallowing dysfunction after primary (chemo) radiation: results of a prospective observational study Radiother Oncol 2012 105 107 114 21907437 10.1016/j.radonc.2011.08.009

[B28] Rancati T Schwarz M Allen AM Feng F Popovtzer A Mittal B Eisbruch A Radiation dose-volume effects in the larynx and pharynx Int J Radiat Oncol Biol Phys 2010 76 S64 S69 20171520 10.1016/j.ijrobp.2009.03.079 PMC2833104

[B29] Raaijmakers E Engelen AM Is sensorineural hearing loss a possible side effect of nasopharyngeal and parotid irradiation? A systematic review of the literature Radiother Oncol 2002 65 1 7 12413668 10.1016/s0167-8140(02)00211-6

[B30] Bhide SA Kazi R Newbold K Harrington KJ Nutting CM The role of intensity-modulated radiotherapy in head and neck cancer Indian J Cancer 2010 47 267 273 20587901 10.4103/0019-509X.64719

[B31] Bhide SA Harrington KJ Nutting CM Otological toxicity after postoperative radiotherapy for parotid tumours Clin Oncol (R Coll Radiol) 2007 19 77 82 17305258 10.1016/j.clon.2006.11.007

[B32] Cacciatore F Napoli C Abete P Marciano E Triassi M Rengo F Quality of life determinants and hearing function in an elderly population: Osservatorio geriatrico campano study group Gerontology 1999 45 323 328 10559650 10.1159/000022113

[B33] Bhandare N Jackson A Eisbruch A Pan CC Flickinger JC Antonelli P Mendenhall WM Radiation therapy and hearing loss Int J Radiat Oncol Biol Phys 2010 76 S50 S57 20171518 10.1016/j.ijrobp.2009.04.096 PMC3319461

[B34] Barker JL Jr Garden AS Ang KK O'Daniel JC Wang H Court LE Morrison WH Rosenthal DI Chao KS Tucker SL Mohan R Dong L Quantification of volumetric and geometric changes occurring during fractionated radiotherapy for head-and-neck cancer using an integrated CT/linear accelerator system Int J Radiat Oncol Biol Phys 2004 59 960 970 15234029 10.1016/j.ijrobp.2003.12.024

[B35] Robar JL Day A Clancey J Kelly R Yewondwossen M Hollenhorst H Rajaraman M Wilke D Spatial and dosimetric variability of organs at risk in head-and-neck intensity-modulated radiotherapy Int J Radiat Oncol Biol Phys 2007 68 1121 1130 17398025 10.1016/j.ijrobp.2007.01.030

[B36] Lee C Langen KM Lu W Haimerl J Schnarr E Ruchala KJ Olivera GH Meeks SL Kupelian PA Shellenberger TD Manon RR Assessment of parotid gland dose changes during head and neck cancer radiotherapy using daily megavoltage computed tomography and deformable image registration Int J Radiat Oncol Biol Phys 2008 71 1563 1571 18538505 10.1016/j.ijrobp.2008.04.013

[B37] Castadot P Geets X Lee JA Christian N Gregoire V Assessment by a deformable registration method of the volumetric and positional changes of target volumes and organs at risk in pharyngo-laryngeal tumors treated with concomitant chemo-radiation Radiother Oncol 2010 95 209 217 20385413 10.1016/j.radonc.2010.03.007

[B38] Zhang T Chi Y Meldolesi E Yan D Automatic delineation of on-line head-and-neck computed tomography images: Toward on-line adaptive radiotherapy Int J Radiat Oncol Biol Phys 2007 68 522 530 17418960 10.1016/j.ijrobp.2007.01.038

[B39] Lee C Langen KM Lu W Haimerl J Schnarr E Ruchala KJ Olivera GH Meeks SL Kupelian PA Shellenberger TD Manon RR Evaluation of geometric changes of parotid glands during head and neck cancer radiotherapy using daily MVCT and automatic deformable registration Radiother Oncol 2008 89 81 88 18707786 10.1016/j.radonc.2008.07.006

[B40] Tsuji SY Hwang A Weinberg V Yom SS Quivey JM Xia P Dosimetric evaluation of automatic segmentation for adaptive IMRT for head-and-neck cancer Int J Radiat Oncol Biol Phys 2010 77 707 714 20231063 10.1016/j.ijrobp.2009.06.012

[B41] Mattiucci GC Boldrini L Chiloiro G D'Agostino GR Chiesa S De Rose F Azario L Pasini D Gambacorta MA Balducci M Valentini V Automatic delineation for replanning in nasopharynx radiotherapy: What is the agreement among experts to be considered as benchmark? Acta Oncol 2013 52 1417 1422 23957565 10.3109/0284186X.2013.813069

[B42] Ho KF Marchant T Moore C Webster G Rowbottom C Penington H Lee L Yap B Sykes A Slevin N Monitoring dosimetric impact of weight loss with kilovoltage (kv) cone beam CT (CBCT) during parotid-sparing imrt and concurrent chemotherapy Int J Radiat Oncol Biol Phys 2012 82 e375 e382 22197229 10.1016/j.ijrobp.2011.07.004

[B43] Geets X Daisne JF Tomsej M Duprez T Lonneux M Gregoire V Impact of the type of imaging modality on target volumes delineation and dose distribution in pharyngo-laryngeal squamous cell carcinoma: Comparison between pre- and per-treatment studies Radiother Oncol 2006 78 291 297 16499982 10.1016/j.radonc.2006.01.006

[B44] Geets X Tomsej M Lee JA Duprez T Coche E Cosnard G Lonneux M Gregoire V Adaptive biological image-guided IMRT with anatomic and functional imaging in pharyngo-laryngeal tumors: Impact on target volume delineation and dose distribution using helical tomotherapy Radiother Oncol 2007 85 105 115 17562346 10.1016/j.radonc.2007.05.010

[B45] Hansen EK Bucci MK Quivey JM Weinberg V Xia P Repeat CT imaging and replanning during the course of IMRT for head-and-neck cancer Int J Radiat Oncol Biol Phys 2006 64 355 362 16256277 10.1016/j.ijrobp.2005.07.957

[B46] Qazi AA Pekar V Kim J Xie J Breen SL Jaffray DA Auto-segmentation of normal and target structures in head and neck CT images: a feature-driven model-based approach Med Phys 2011 38 6160 6170 22047381 10.1118/1.3654160

[B47] Whitfield GA Price P Price GJ Moore CJ Automated delineation of radiotherapy volumes: Are we going in the right direction? Br J Radiol 2013 86 20110718 23239689 10.1259/bjr.20110718 PMC3615399

[B48] van de Water TA Bijl HP Westerlaan HE Langendijk JA Delineation guidelines for organs at risk involved in radiation-induced salivary dysfunction and xerostomia Radiother Oncol 2009 93 545 552 19853316 10.1016/j.radonc.2009.09.008

[B49] Pacholke HD Amdur RJ Schmalfuss IM Louis D Mendenhall WM Contouring the middle and inner ear on radiotherapy planning scans Am J Clin Oncol 2005 28 143 147 15803007 10.1097/01.coc.0000143847.57027.16

[B50] Warfield SK Zou KH Wells WM Simultaneous truth and performance level estimation (staple): an algorithm for the validation of image segmentation IEEE Trans Med Imaging 2004 23 903 921 15250643 10.1109/TMI.2004.828354 PMC1283110

[B51] Dice LR Measures of the amount of ecologic association between species Ecology 1945 26 297 302

[B52] Zijdenbos AP Dawant BM Margolin RA Palmer AC Morphometric analysis of white matter lesions in MR images: method and validation IEEE Trans Med Imaging 1994 13 716 724 18218550 10.1109/42.363096

[B53] Bzdusek KBD Pekar V Peters J Schadewaldt N Schulz H Vik T Smart Probabilistic Image Contouring Engine (SPICE) Available at: http://www.healthcare.philips.com/pwc_hc/main/shared/Assets/Documents/Ros/452296286221_SPICE_WP_LR.pdf

[B54] Chao KS Bhide S Chen H Asper J Bush S Franklin G Kavadi V Liengswangwong V Gordon W Raben A Strasser J Koprowski C Frank S Chronowski G Ahamad A Malyapa R Zhang L Dong L Reduce in variation and improve efficiency of target volume delineation by a computer-assisted system using a deformable image registration approach Int J Radiat Oncol Biol Phys 2007 68 1512 1521 17674982 10.1016/j.ijrobp.2007.04.037

[B55] Sims R Isambert A Gregoire V Bidault F Fresco L Sage J Mills J Bourhis J Lefkopoulos D Commowick O Benkebil M Malandain G A pre-clinical assessment of an atlas-based automatic segmentation tool for the head and neck Radiother Oncol 2009 93 474 478 19758720 10.1016/j.radonc.2009.08.013

[B56] Daisne JF Blumhofer A Atlas-based automatic segmentation of head and neck organs at risk and nodal target volumes: a clinical validation Radiat Oncol 2013 8 154 23803232 10.1186/1748-717X-8-154 PMC3722083

[B57] Teguh DN Levendag PC Voet PW Al-Mamgani A Han X Wolf TK Hibbard LS Nowak P Akhiat H Dirkx ML Heijmen BJ Hoogeman MS Clinical validation of atlas-based auto-segmentation of multiple target volumes and normal tissue (swallowing/mastication) structures in the head and neck Int J Radiat Oncol Biol Phys 2011 81 950 957 20932664 10.1016/j.ijrobp.2010.07.009

[B58] Speight RKE Prestwich R Sen M Lindsay R Harding R Sykes J Evaluation of atlas based auto-segmentation for head and neck target volume delineation in adaptive/replan IMRT J Geophys Res 2014 489 012060 doi:101088/1742-6596/489/1/012060

